# A rational framework for evaluating the next generation of vaccines against *Mycobacterium avium* subspecies *paratuberculosis*

**DOI:** 10.3389/fcimb.2014.00126

**Published:** 2014-09-09

**Authors:** John P. Bannantine, Murray E. Hines, Luiz E. Bermudez, Adel M. Talaat, Srinand Sreevatsan, Judith R. Stabel, Yung-Fu Chang, Paul M. Coussens, Raúl G. Barletta, William C. Davis, Desmond M. Collins, Yrjö T. Gröhn, Vivek Kapur

**Affiliations:** ^1^Infectious Bacterial Diseases USDA-ARS, National Animal Disease CenterAmes, IA, USA; ^2^Tifton Veterinary Diagnostic and Investigational Lab, The University of GeorgiaTifton, GA, USA; ^3^Departments of Microbiology and Biomedical Sciences, Oregon State UniversityCorvalis, OR, USA; ^4^Department of Pathobiological Sciences, University of Wisconsin-MadisonMadison, WI, USA; ^5^Department of Food Hygenie, Cairo UniversityCairo, Egypt; ^6^Veterinary Population Medicine Department, University of MinnesotaMinneapolis, MN, USA; ^7^Department of Population Medicine and Diagnostic Sciences, College of Veterinary Medicine, Cornell UniversityIthaca, NY, USA; ^8^Department of Animal Science, Michigan State UniversityLansing, MI, USA; ^9^School of Veterinary Medicine and Biomedical Sciences, University of NebraskaLincoln, NE, USA; ^10^Department of Veterinary Microbiology, Washington State UniversityPullman, WA, USA; ^11^AgResearchWallaceville, New Zealand; ^12^Department of Veterinary and Biomedical Sciences, Pennsylvania State UniversityUniversity Park, PA, USA

**Keywords:** Johne's disease, *Mycobacterium*, vaccines, attenuated, transposons, animal models, genomics

## Abstract

Since the early 1980s, several investigations have focused on developing a vaccine against *Mycobacterium avium* subspecies *paratuberculosis* (MAP), the causative agent of Johne's disease in cattle and sheep. These studies used whole-cell inactivated vaccines that have proven useful in limiting disease progression, but have not prevented infection. In contrast, modified live vaccines that invoke a Th1 type immune response, may improve protection against infection. Spurred by recent advances in the ability to create defined knockouts in MAP, several independent laboratories have developed modified live vaccine candidates by transpositional mutation of virulence and metabolic genes in MAP. In order to accelerate the process of identification and comparative evaluation of the most promising modified live MAP vaccine candidates, members of a multi-institutional USDA-funded research consortium, the Johne's disease integrated program (JDIP), met to establish a standardized testing platform using agreed upon protocols. A total of 22 candidates vaccine strains developed in five independent laboratories in the United States and New Zealand voluntarily entered into a double blind stage gated trial pipeline. In Phase I, the survival characteristics of each candidate were determined in bovine macrophages. Attenuated strains moved to Phase II, where tissue colonization of C57/BL6 mice were evaluated in a challenge model. In Phase III, five promising candidates from Phase I and II were evaluated for their ability to reduce fecal shedding, tissue colonization and pathology in a baby goat challenge model. Formation of a multi-institutional consortium for vaccine strain evaluation has revealed insights for the implementation of vaccine trials for Johne's disease and other animal pathogens. We conclude by suggesting the best way forward based on this 3-phase trial experience and challenge the rationale for use of a macrophage-to-mouse-to native host pipeline for MAP vaccine development.

## Introduction

Johne's disease is caused by *Mycobacterium avium* subspecies *paratuberculosis* (hereafter referred to as MAP), an acid-fast bacillus that can be distinguished from other closely related mycobacteria by its unique requirement for the mycobactin J siderophore in artificial culture media (Merkal and Curran, 1974). This major veterinary pathogen can infect many species of animals (Whittington et al., 2011), but its impact is felt most profoundly on commercial ruminant livestock such as cattle, sheep, goats and deer. In the United States (US) alone, current economic loses to the dairy industry are unknown, but previously was estimated at between 200 million and 1.5 billion dollars annually (Stabel, 1998). Almost three decades ago, the prevalence of Johne's disease in US dairy cattle was estimated at between 3 and 18% based on slaughterhouse surveys (Chiodini and Van Kruiningen, 1986; Merkal et al., 1987). A more recent national level serological survey conducted by the National Animal Health Monitoring System (NAHMS) in 2007 suggested MAP prevalence on US dairy farms had risen above 30%. Unfortunately, this percentage continues to climb with the passage of time and implementation of more sensitive diagnostic tests. The most recent US dairy herd prevalence estimates are as high as 90% (Lombard et al., 2013) and New Zealand farmed deer herd estimates are at 59% (Stringer et al., 2013a). Collectively, these data suggest that MAP infection has long been endemic in the US and most likely across the world wherever dairy cows, sheep, goats and deer are intensively raised.

Vaccination against MAP infection has long been thought to be the best intervention strategy for this chronic and debilitating disease that is difficult to diagnose and slow to manifest. Animals actively shed large quantities of MAP before being diagnosed or exhibiting clinical signs—resulting in a transmission cycle that is very difficult to interrupt using traditional management strategies alone. Sub clinically infected animals transmit disease while appearing healthy and remaining undetectable by culture or PCR based approaches since these animals often shed MAP in a sporadic or intermittent manner. In stark contrast to the subversive “trickle and stealth” shedding pattern of MAP frequently observed in subclinical animals, there exist symptomatic, high shedder animals that excrete prodigious levels of organisms (up to 10^8^ CFU per gram) in their feces, which provide the source of significant on-farm contamination (Pradhan et al., 2011). Diagnostic tests for Johne's disease are improving, but accurate detection of all infected animals, especially those that are early in infection and transmitting the organism within a herd, is still not possible. This fact makes test and cull strategies ineffective, except when targeting only high-shedding animals (Lu et al., 2008; Bastida and Juste, 2011). Therefore, it is widely recognized that unless animals can be detected early during infection, vaccination remains the best hope for controlling and preventing Johne's disease.

The ideal vaccine would completely prevent infection and/or promote protective immunity thus blocking both horizontal and vertical transmission. The current vaccines for Johne's disease fall far short of this high standard. MAP vaccines have been shown to be effective at lowering fecal shedding levels (Kalis et al., 2001; Faisal et al., 2013a), tissue colonization (Sweeney et al., 2009) or clinical disease incidence (Stringer et al., 2013b), but do not completely eliminate all three. Subunit vaccines against MAP are likely to obviate some of the shortcomings of whole-cell vaccines, such as severe inflammation and granuloma formation at the injection site. However, subunit vaccines that have been tested thus far have yielded incomplete protection results in murine models of infection (Koets et al., 2006; Stabel et al., 2012) and even when combinations of proteins are used in calves and goats (Koets et al., 2006; Kathaperumal et al., 2008, 2009). For example, MAP was colonized in the lymph node and spleen at similar levels in control and vaccinate mice using a protein cocktail (Stabel et al., 2012). Similarly, there were no significant MAP burden differences in the liver and mesenteric lymph node at 8 weeks post-challenge when immunizing mice with a 74 kDa polyprotein, but there was a significant difference in the liver (Chen et al., 2008). However, additional studies using more animals, longer term to monitor protection (1–3 years) and field trials are needed. Unfortunately subunit vaccines are expensive to produce whereas the manufacture of attenuated mutants is low in cost and easy to produce. These considerations are even more important for food animal health. Thus, there are several considerations, but research in this area demonstrates the slow yet steady progress in vaccine development for Johne's disease.

Efforts to improve upon current vaccine formulations have long been hampered by the lack of standardized challenge models and the high costs associated with trials conducted in natural ovine, caprine, deer or bovine hosts. Progress is however being made, and standardized animal models (mouse, goat, cow) for Johne's disease have been developed by a group of international investigators under the auspices of the Johne's Disease Integrated Program (JDIP), a USDA-funded research consortium (Hines et al., 2007) to help facilitate comparisons between different vaccine trials. However, the costs and logistical challenges of MAP vaccine trials, particularly in the natural hosts, remain a major concern. And even with an efficacious vaccine, it is remotely possible that MAP could be maintained within a herd due to vertical transmission as noted in a recent vaccine modeling study (Lu et al., 2013). Finally, there are negative implications of JD vaccination on both Johne's disease and bovine TB diagnostics as will be discussed further below.

Consistent with what is known about other intracellular pathogens, a MAP vaccine that drives the immune response to a proinflammatory Th1 profile and prevents a shift to the humoral Th2 response, maybe more effective in delaying progression of the disease to a clinical state (Coussens, 2004; Stabel, 2010). However, it has recently been suggested that humoral immunity may also be important against *M. tuberculosis* infection (Achkar and Casadevall, 2013). Nonetheless, current dogma suggests a late humoral response is not a protective immune response against Johne's disease. The focus of the JDIP vaccine studies was on live attenuated mutants rather than subunit or DNA vaccines because they are more likely to generate the beneficial Th1 immune response, which is considered the most protective against mycobacterial infections (Stabel, 2000; Hostetter et al., 2002). The cause for the switch from Th1 to Th2 immune responses in Johne's disease is unknown, however mathematical modeling of the bovine immune response to MAP suggests it may be attributed to extracellular MAP that persists outside of macrophages (Magombedze et al., 2014). Although these inferences need to be carefully tested experimentally, it is possible a mutant strain deficient in this capability might be the critical factor to an efficacious vaccine for Johne's disease.

Despite significant recent advances in the ability to make defined mutants, there have been only a limited number of trials testing live attenuated vaccines against MAP. These have been largely limited to *in vitro* studies or trials in the mouse (Scandurra et al., 2009; Settles et al., 2014), and except in a few cases (Scandurra et al., 2010; Faisal et al., 2013b), reflect a striking absence of robust and cost effective strategies for evaluating vaccine candidates in the relevant natural ruminant host. To help address this unmet need, and establish a rational framework based for the evaluation of novel vaccine candidates against MAP, we adopted an inclusive and collaborative approach that may also serve as a guide for future vaccine evaluation trials for MAP and other major animal pathogens. We here present the state-of-the-field summary for vaccination against Johne's disease, describe current candidate live attenuated mutants of MAP, and present an overview of the JDIP three-phase vaccine trial and lessons learned.

## MAP vaccines: what is available?

It is paradoxical that vaccination against MAP has not been widely used in the US given the high prevalence of Johne's disease in this country. Currently, there are at least three commercial Johne's disease vaccines worldwide, but only one (Mycopar®) approved vaccine for use in the US (Bastida and Juste, 2011). Silirum® is produced by Zoetis and was used as the control vaccine in the JDIP mouse and goat trials discussed below. Mycopar® is manufactured by Boehringer Ingelheim Vetmedica Inc. whereas Gudair® is produced by CZ Veterinaria in Spain and is being used in countries with large sheep populations (Bastida and Juste, 2011). Importantly, all three consist of an inactivated whole-cell preparation of either *M. avium* subspecies *avium* or subspecies *paratuberculosis*. As summarized by Bull et al. live attenuated strains were used in the 1960s and 1970s and then concerns about their use in the 1980s forced a shift to using killed vaccine formulations (Bull et al., 2013). Live vaccine strains can now be revisited with the technology used to create defined mutations.

What does the scientific literature report about the efficacy of these commercial MAP vaccines? In three dairy herd operations with Johne's disease, investigators showed that fewer Mycopar® vaccinated cattle had detectable fecal culture positive results compared with controls (Knust et al., 2013). This same vaccine also showed partial protection in a goat challenge model (Faisal et al., 2013a). However, the inactivated strain used in the Mycopar® formulation is not MAP, but an isolate called strain 18 that is nonetheless a member of the *M. avium* species. The manufacturer data sheet still lists Mycopar® as an “inactivated *M. paratuberculosis* bacterium suspended in oil.” Interestingly, this parallelism occurs with the suboptimal response of BCG, derived from the related species *M. bovis*, as a vaccine against tuberculosis (Mangtani et al., 2014). Gudair consists of heat-killed MAP 316F and is designed for use in sheep and goats according to the manufacturer. Gudair® has been shown to be an effective vaccine in Merino sheep (Reddacliff et al., 2006). Vaccination not only delayed the onset of fecal shedding, but reduced mortality attributed to Johne's disease by 90% in the 200 vaccinated sheep. Silirum® is a killed strain of MAP, also related to 316F, that has been tested in Australian cattle. This vaccine has recently been shown to reduce prevalence of clinical disease, as measured by lymph node pathology and fecal culture at slaughter, in farmed deer in New Zealand (Stringer et al., 2013b). Currently, there are no subunit, live or DNA vaccines commercially available against Johne's disease.

## Construction of attenuated MAP mutants

Although attenuated MAP, generated by repeated subculture of the strain, had been used as a live vaccine long ago (Saxegaard and Fodstad, 1985), no defined mutants of MAP had been tested as a vaccine simply because no method was available for their construction. In 1995, the first concerted effort to develop the genetics of MAP was performed in Raúl Barletta's laboratory at the University of Nebraska (Foley-Thomas et al., 1995). Studies in his laboratory demonstrated for the first time that MAP could be transformed with foreign DNA as well as transfected with bacteriophage DNA. Furthermore, they found that MAP could be productively infected with the mycobacteriophage TM4 and later demonstrated the use of a thermosensitive derivative of TM4 to generate the first transposon mutant library (Harris et al., 1999). This methodological advance was adapted by other laboratories to create their own transposon mutant banks (Cavaignac et al., 2000; Shin et al., 2006). Phage-mediated techniques are now being applied to construct directed knockout mutations in MAP using allelic exchange. This technique worked well in *M. tuberculosis*, but the allelic exchange efficiency was low in MAP until investigators at Washington State University tweaked the protocol to increase efficiencies to generate *ΔrelA, Δlsr2*, and *ΔpknG* knockout strains (Park et al., 2008a). Now robust enough to be performed in other laboratories, this method enabled a more strategic approach to obtaining the precise knockout of a specific virulence gene rather than depend on random transposon mutagenesis and screening approaches to find a suitable knockout to try as a vaccine. The *ppiA* (Scandurra et al., 2010), *relA, lsr2, pknG* (Park et al., 2008a)*, leuD, mpt64*, and *secA2* genes (Chen et al., 2012) have all been successfully knocked out using this method. Each of these mutants has also been evaluated as a vaccine candidate as discussed in the next section.

## Previous MAP vaccine studies

MAP vaccination studies have been thoroughly summarized for cattle, sheep and goats by examining pathological, epidemiological and production effects of vaccination (Bastida and Juste, 2011). This meta-analysis revealed that while vaccination is useful in limiting microbial contamination and production losses, most of the vaccines are similar in content and preparation. These findings have recently been reinforced by the reduction of fecal contamination in three dairy herds using a killed vaccine (Knust et al., 2013). The possibility exists that new generations of vaccine formulations, including live attenuated, may further improve on the killed vaccine preparations currently being used. This section summarizes the pertinent literature on MAP vaccination as it relates to the JDIP trials.

Subunit vaccines have been delivered using heterologous hosts such as *Salmonella* (Chandra et al., 2012), or *Lactobacillus* (Johnston et al., 2014). Only two DNA vaccines against MAP have been tested in mice. One used expression library DNA immunization of Balb/cJ mice and challenged with a recently isolated bovine MAP strain 6112 (Huntley et al., 2005). Four pools of 108 clones each protected mice from colonization of spleen and mesenteric lymph nodes. The second study tested a cocktail of five genes encoding antigen 85A, B, and C along with superoxide dismutase and 35-kDa protein by intramuscular injection of C57/BL6 (Park et al., 2008b). There was a significant reduction in colonization of the liver and spleen of mice immunized with this cocktail. The subunit vaccines from both studies induced a Th1 immune response as measured by interferon gamma; however, none of these DNA formulations were tested in cattle or other ruminant hosts.

Several *MAP* vaccine candidates have been analyzed in isolation making it difficult to directly compare candidates between studies. Some have been bench marked against a commercially available vaccine (Faisal et al., 2013a; Hines et al., 2014) while other studies lack even that as a reference. As mentioned, all commercial vaccine formulations consist of a killed whole-cell preparation of MAP in an oil emulsion, but there are many other types of vaccines. Still other vaccine formulations included live *M. avium* bacteria, but it is not clear if or how they were attenuated (Begg and Griffin, 2005). In the current JDIP vaccine trial, we took a different approach in identifying a viable, yet attenuated vaccine strain, reasoning that a live strain will maintain a proinflammatory Th1 response (Stabel, 2000; Coussens, 2004). Th1-associated cytokines including interferon gamma, interleukin-2 (IL-2), IL-12 and tumor necrosis factor alpha were measured after antigen stimulation of cultured peripheral blood monocytes in these studies.

While there are no published bovine vaccine trials in Johne's disease, there are a number of caprine and ovine vaccine trials. Furthermore, a number of MAP vaccine studies in mice have been reported, but they are too numerous to adequately be summarized here. Importantly, only a few studies that began using the mouse model were further evaluated in a ruminant host (Scandurra et al., 2010; Park et al., 2011) and none using the number of mutant strains in the JDIP trials. This was a key component in the design of the JDIP vaccine trial. Using the data described in this special topics issue, we can now assess the predictive value of the mouse trial in obtaining good candidates for the ruminant host.

### Attenuated mutants in the JDIP vaccine study

What was known about the mutants prior to enrollment in the JDIP vaccine study? Some of the mutants remain unpublished or have only recently been submitted for publication, however, others have been described prior to the JDIP trials. Information concerning all mutants included in the JDIP trials is summarized in Table 1. The first MAP mutants created by allelic exchange produced directed knockouts of *lsr2, relA*, and *pknG* mutants in 2008 (Park et al., 2008a) and all three were included in the JDIP trial (Table 1). These three genes were selected based on the virulence properties of their orthologs in *Mycobacterium tuberculosis* and *Mycobacterium bovis*. Lsr2 is a cytosolic protein implicated in cell wall lipid biosynthesis and antibiotic resistance in *M. smegmatis* (Chen et al., 2006; Colangeli et al., 2007). Protein kinase G, encoded by *pknG*, is secreted by *M. tuberculosis* and *M. bovis* within the phagosome of macrophages and is thought to inhibit phagolysosomal fusion (Walburger et al., 2004). RelA in *M. tuberculosis* is involved in the stringent response that is activated in nutrient limiting conditions. Specifically, RelA in *M. tuberculosis* synthesizes the hyperphosphorylated guanine nucleotides that accumulate in nutrient limiting conditions and inactivating this gene severely reduced long-term survival in mice (Dahl et al., 2003).

**Table 1:** **Transposon mutant vaccine candidates of MAP enrolled in the JDIP vaccine trials**.

**Institution^a^**	**Blinded code^b^**	**Location of insertion^c^**	**MAP strain background^d^**	**Moved to**		**References**
				**Phase II?^e^**	**Phase III?^f^**	
USDA-ARS-WRRC	311	MAP0482	Goat strain 43432-02	No	No	McGarvey, unpublished
Washington State University	312	MAP1047 (*relA*)	K-10	No	No	Park et al., 2008a
Washington State University	313	MAP3893c (*pknG*)	K-10	No	No	Park et al., 2008a
Washington State University	314	MAP0460 (*lsr2*)	K-10	No	No	Park et al., 2008a
University of Nebraska	315	MAP1566	K-10	Yes	Yes	Rathnaiah et al., in review
University of Nebraska	316	MAP3695 and *fadE5*	K-10	Yes	Yes	Rathnaiah et al., in review
University of Nebraska	317	MAP0460 (*lsr2*)	K-10	Yes	No	Rathnaiah et al., in review
University of Nebraska	318	MAP0282c and 0283c	K-10	Yes	Yes	Rathnaiah et al., in review
University of Nebraska	319	MAP1566	K-10	Yes	Yes	Rathnaiah et al., in review
University of Nebraska	320	MAP2296c and 2297c	K-10	Yes	No	Rathnaiah et al., in review
University of Nebraska	321	MAP1150c and 1151c	K-10	Yes	No	Rathnaiah et al., in review
AgResearch NZ	322	MAP1566	strain 989	No	No	Scandurra et al., 2010
AgResearch NZ	323	MAP0011 (*ppiA*)	K-10	No	No	Scandurra et al., 2010
University of Wisconsin	324	MAP0997c (*kdpC*)	ATCC19698	No	No	Shin et al., 2006
University of Wisconsin	325	MAP3006c (*lipN*)	K-10	No	No	
University of Wisconsin	326	MAP3963 (*umaA1*)	ATCC19698	No	No	Shin et al., 2006
University of Wisconsin	327	MAP4287c	K-10	No	No	
University of Wisconsin	328	MAP1242 (*pstA*)	ATCC19698	No	No	
University of Wisconsin	329	MAP2408c (*fabG2_2*)	ATCC19698	Yes	Yes	Shin et al., 2006
University of Wisconsin	330	MAP1719c	ATCC19698	No	No	
University of Wisconsin	331	MAP1872c (*mbtH_2*)	ATCC19698	No	No	Kabara and Coussens, 2012
University of Wisconsin	332	MAP4288 (*lpqP*)	ATCC19698	No	No	

a *The location of the laboratory where the mutant(s) was constructed*.

b *The stains were cultured and blinded at Penn State University prior to shipment to the testing labs*.

c *The MAP locus where the transposon inserted. If two genes are listed, the transposon is inserted in the intergenic region between the two. If the gene has been named, it is shown in parenthesis*.

d *The parental strain of MAP used to create the mutation*.

e *Indicates if the mutant strain was moved forward into the phase II (mouse) trial*.

f *Indicates if the mutant strain was moved forward into the phase III (goat) trial*.

Two of these MAP mutants, Δ*pknG* and Δ*relA*, were later tested as vaccine candidates in cultured macrophages, calves and kid goats (Park et al., 2011). Although both were attenuated in bovine macrophages compared to the wild-type strain on day 6, it was shown that Δ*relA* was the better vaccine candidate since no MAP was found in the tissues of calves vaccinated with the *relA* mutant. Furthermore, vaccination with the *pknG* mutant did not inhibit challenge with MAP in the kid goats as the challenge bacteria were present in high numbers in all 9 tissues evaluated, whereas the *relA* mutant vaccinates were free of the challenge inoculum in 8 of the 9 tissues taken from kid goats (Park et al., 2011). However, both mutants induced effector memory T cells similar to the wild type strain. Although Δ*lsr2* was included in the study, further experiments were needed to assess the *in vivo* survival of Δ*lsr2* because of fungus problems in readout cultures (see Park et al., 2014, this issue). Of interest, a second mutant containing an *lsr2* transposon insertion was developed by another laboratory and included in the JDIP study and was coded 317 (Table 1). A report on this mutant has just been submitted publication, and it was evaluated in the mouse challenge model in phase II but not the phase III JDIP trial. Another interesting observation was that three independent investigators submitted mutants with insertions in the same gene, MAP1566, which encodes a hypothetical protein (Li et al., 2005). This is surprising because the MAP genome contains over 4300 targets for transposon insertion, yet of the 22 mutants enrolled in this study 3 are in the same target (MAP1566) and another two are in *lsr2*.

One of the three ΔMAP1566 mutants along with the *ppiA* mutant, coded as 322 and 323, respectively, in Table 1, were analyzed in macrophages, mice, and goats (Scandurra et al., 2010). All three model systems demonstrated that ΔMAP1566 was more attenuated than the *ppiA* mutant. However, macrophages produced less IL-10 when infected with the *ppiA* mutant and it persisted longer in mice. In the goat experiment, the challenge strain was not cultured from any of the tissues in goats vaccinated with ΔMAP1566, whereas 15% of the tissues taken from the Δ*ppiA* immunized goats were positive by Bactec 12B culture for the challenge strain (Scandurra et al., 2010). Collectively, these data suggest that the ΔMAP1566 strain is a better vaccine than Δ*ppiA*.

Other mutants that were shown to be attenuated in mice prior to testing in the JDIP vaccine trial include *kdpC, pstA, umaA1*, and *fabG2_2* (Shin et al., 2006). The *pstA* gene encodes a large (12 kb) non-ribosomal synthetase protein involved in glycopeptidolipid biosynthesis and was later shown to contribute to biofilm formation and invasion of the calf intestine (Wu et al., 2009). The *umaA1* gene codes for a mycolic acid synthase that was studied in *M. tuberculosis* (Yuan et al., 1995). The *kdpC* mutant showed significantly lower colonization levels in the liver and intestine of mice at all time points, compared to the wild-type and also displayed less granulomatous inflammation (Shin et al., 2006). The *pstA, umaA1*, and *fabG2_2* mutants showed reduced bacterial colonization of the mouse intestine at later time points. Finally, a manuscript is in preparation describing the mutants constructed at the University of Nebraska (Table 1). Among this group are transposon insertions between genes (intergenic) rather than within a gene.

A subset of the mutants in the JDIP study was examined for ability to regulate host cell apoptosis of macrophages (Scandurra et al., 2010; Kabara and Coussens, 2012). Apoptosis is an important tool in the fight against mycobacterial infections as apoptotic bodies are taken up by other antigen presenting cells. Mycobacteria contained in apoptotic bodies are destroyed and their antigens presented to immune cells. The *lsr2* knockout was most similar to the wild-type in terms of controlling apoptosis among the mutants tested, indicating that *lsr2* is not involved in this process. However, a Tn*5367* insertion in MAP1872c was not able to prevent apoptosis, indicating that this gene may be involved in regulating this host cell process. The gene encodes an iron acquisition protein (Zhu et al., 2008), which is an important process for MAP survival in the host. The other mutants tested were not significantly different from the uninfected control, which demonstrated the level of spontaneous apoptosis in culture. A second study examined apoptosis in bovine macrophages (Scandurra et al., 2010) and two of the mutants overlapped between that study and the Kabara and Coussens study. These mutants included the *ppiA* knockout (code 323 in Table 1) and MAP1566 (code 322). While neither of these mutants demonstrated control of apoptosis in the Kabara and Coussens study, ΔMAP1566 showed a significant reduction in apoptosis compared to the wild type strain in the Scandurra et al. study. The *ppiA* mutant was inconclusive since macrophages from the two cows examined gave divergent results (Scandurra et al., 2010). When tested independently all these mutants showed promise as a vaccine candidate for Johne's disease. So we developed a strategy to directly compare all of these mutants in the same vaccine trial through the JDIP research consortium.

### Interference with diagnostic testing for TB or johne's disease

Although it has been suggested there might be an added benefit to vaccinate against MAP for cross protection with TB (Perez De Val et al., 2012), it is generally seen as a negative due to interference with diagnostic testing for TB, a highly regulated animal disease. In fact, a primary reason why whole cell vaccines against Johne's disease are not routinely used in the United States is due to the interference with diagnostic tests for both Johne's disease and particularly bovine TB. This same reason is why vaccination was also reserved only for farms in the Netherlands with high incidence of Johne's disease (Muskens et al., 2002). Immunization with Mycopar® will interfere with Johne's diagnostic tests, particularly the IFN-γ test, but not with the comparative cervical skin test which was 100% specific within the first year of vaccination (Stabel et al., 2011). In addition, *M. bovis* serological tests, which included the ESAT-6, CFP-10, and MPB83 antigens, were also negative in MAP vaccinated calves. Likewise, an immediate strong and long-lasting MAP-specific IFN-γ response was observed in two herds vaccinated with a heat-killed vaccine (Muskens et al., 2002). Caution must be used if administering Gudair® to cattle since it appears to lower the sensitivity of the bovine TB skin test (Coad et al., 2013). Dairy herds vaccinated with Silirum® showed that the comparative intradermal test (CIT) had low cross reactivity; however the single bovine intradermal tubculin test would result in 5–6% false positive results for TB (Garrido et al., 2013). These results are similar with MAP vaccinated goats (Chartier et al., 2012). Thus, the CIT test can be considered useful if evaluating TB in a MAP vaccinated herd. This finding paves the way for live attenuated vaccines for MAP. Perhaps even more encouraging are the novel biomarkers identified from proteomics of both the host and pathogen that suggest peptides in circulation may overcome the issue of cross-reactivity (Seth et al., 2009). However, even if producers had to deal with a low level of false positive results for TB infection, we believe that if a strong live vaccine were developed that could prevent infection and enable effective disease control, the vaccine would be widely implemented.

## Conception of the JDIP vaccine trial

It was against this background that the idea for establishing independent testing labs to evaluate all the available vaccine candidates was conceived. This multi-institutional study was based on the following principles. Through the formation of a research consortium on Johne's disease, which was funded by the USDA-NIFA from 2002 to 2011, this governing body was successful in setting up a world-wide search for the best performing vaccine candidates. The idea behind this project was to combine the resource and intellect of JDIP with the financial resource of USDA-APHIS-VS to identify the best possible attenuated vaccine available. On January 12, 2008 a meeting was held in Chicago, Illinois to devise a coordinated effort to test attenuated vaccine candidates submitted by Johne's disease researchers. Although a few vaccine trials have tested individual mutants in macrophages, mice and goats, no trial of this scope using 5 or more mutants tested in parallel has ever been performed.

### Trial design

To begin, investigators around the world were asked to submit their best-attenuated mutants, along with any efficacy data generated in their labs, to the JDIP study. A multi-institutional universal material transfer agreement was drafted and finalized enabling all investigators to submit their vaccine candidate(s) to Penn State University (PSU) while at the same time, protecting intellectual property rights of the investigator. PSU cultured each strain to the specified optical density and coded each strain for blinding before sending onto specific labs to perform the efficacy trials.

Three gates were decided upon which the mutants had to pass through at each phase of the study. The first gate measured survival of mutants in primary bovine macrophages, where the most attenuated strains were considered the best candidates. The second gate was protection from colonization by challenge bacteria in the liver and spleen of mice and the third gate was protection from colonization using the goat challenge model (Hines et al., 2007). A triage process had to be implemented at each gate due to funding limitations, but we acknowledge it would have been ideal to test all the vaccine strains in all three trials.

Five investigators from the United States and New Zealand responded to the JDIP request and submitted their attenuated MAP mutants. It is noteworthy that directed or random knockouts in MAP are not trivial to obtain and hence, only a few investigators had possession of such mutants. A total of 22 transposon-marked mutants were submitted (Table 1). Interestingly, among the 22 mutants, a few had insertions in the same gene, but were submitted by different investigators. As mentioned above, MAP1566 insertions were present in 3 strains, 2 of them from the same lab contained insertions in different locations within the gene. Also 2 mutants submitted by independent labs were MAP0460 knockouts. Given the number of potential gene targets in this organism (>4300), this duplication was unexpected, but provided some interesting built-in comparisons.

### Vaccine candidate selection

The initial criteria for candidate vaccine selection were based on data obtained by the investigator who constructed the mutant. Each investigator determined independently the suitability of their mutant. In some cases these results were published, but regardless, the net was cast broad and wide to obtain as many MAP mutants as possible for testing. However, there were some minimal qualifications. To enter the first trial each mutant (1) must be viable *in vitro* (no sonicated extracts or heat-inactivated preps) and (2) growth rate in broth cultures must be similar to wild-type MAP. The mutant strains were sent to PSU under a unique pan-institutional material transfer agreement that allowed the submitting investigators to retain full intellectual property. Each mutant strain received was tested for culture, coded to blind testing labs and quality control tested prior to shipping to the testing labs. For example, the PSU lab confirmed a lack of contamination in the culture by PCR and plating on blood agar and/or BHI before shipping. Therefore, after receiving the strains, at least 60 days were needed to prepare each strain for shipment. Five submitted strains were not shipped to the phase I testing labs for the following reasons. One showed incredibly slow and anemic growth in both liquid and solid culture, regardless of several attempts. For two others, the reference colony forming units (CFU) was not obtainable by the end of phase I. And the remaining two arrived after the due date for submission. Although PSU started to grow them regardless, there was not enough time to include them in this initial trial. A general summary of each trial is listed below.

### Phase I: survival in primary bovine macrophages

The single goal of this experiment was to test survival in primary bovine macrophages because attenuation in this environment can infer potential vaccine candidates. Primary bovine macrophages were cultured and infected in two laboratories, one at the University of Minnesota (UMN) and the other at the University of Wisconsin (UW). In order for these labs to start their macrophage studies, a final CFU number was needed from PSU to infect at the correct multiplicity of infection. Once the cells reached OD_600_ = 0.50, the PSU lab determined the reference CFU and started to regrow the strains for shipment. The manuscript describing the results of this study was just published (Lamont et al., 2014).

Colony counts from cultures over time in macrophages were obtained to give the slope (growth over time). Basically, a negative slope demonstrated attenuation because it meant there were less viable bacteria recovered from bovine macrophages as time increased (Lamont et al., 2014). When examining the data by slope alone, it was evident which mutants had a negative slope and which did not, but when looking at the individual CFU data, it was clear that considering slope alone was misleading. Therefore, JDIP solicited the Michigan State University (MSU) lab to perform an apoptosis study of the mutants. Some mutants were not included in this study because of the staggered nature in which they were received or the lack of permission to include the mutant strain in a publication. Most of the strains were sent to the MSU lab for the apoptosis experiment (Kabara and Coussens, 2012). Some of the mutants arrived several months late, and at that point, JDIP needed the data from the study to help make a decision on which mutant strains to go forward in the mouse trial.

Results of the experiments from each trial were sent directly to Cornell University for data analysis. Slope of the growth in macrophages was used to compare mutants. The results showed subtle changes in survival of the mutant strains (manuscript in preparation). Once the analysis was complete and decisions made about which strains would advance to the next phase, the blind was decoded and recoded for the next experiment.

### Phase II: the mouse trial

This experiment was designed to test the remaining 8 attenuated vaccine strains in the mouse challenge model. The readout was colonization (CFU) of the liver and spleen from vaccinated and control mice (Bannantine et al., 2014). Silirum® was the commercial vaccine run in parallel for this trial. Each mouse received 10^5^ CFU of the live attenuated vaccine strain in 0.5 ml PBS by intraperitoneal injection.

The persistence of the vaccine candidates was measured at 6, 12, and 18 weeks post-vaccination. Only strains 320, 321, and 329 colonized both the liver and spleens up until the 12-week time point. The remaining five mutants showed no survival in those tissues, indicating their complete attenuation in the mouse model. The vaccine strains demonstrated different levels of protection based on MAP colonization in liver and spleen tissues at 12 and 18 weeks post-vaccination. Based on total MAP burden in both tissues at both time points, strain 315 (MAP1566::Tn*5370*) was the most protective whereas strain 318 (intergenic Tn*5367* insertion between MAP0282c and MAP0283c) had the most colonization (Bannantine et al., 2014). Mice vaccinated with an undiluted commercial vaccine preparation displayed the strongest antibody responses as well as enlarged spleens.

The effect of persistence of the three vaccine strains in mice is unclear. However, it is clear from the goat trial described below that the vaccine strains did not persist indefinitely as only the challenge strain was identified by PCR analysis at the 2 month time point (Hines et al., 2014). It would have been interesting to see if the *pknG* mutant was persistent in the JDIP goat study, but that mutant was not included in phase III of the trial due to triage after the macrophage study (Lamont et al., 2014). Significantly the *relA* mutant, that also showed attenuation in macrophages, did not make it through to the last phases of the trial, even though a preliminary study in goats showed it was immune eliminated. The immune response elicited by the mutant limited infection with MAP under experimental challenge conditions (Park et al., 2011). A further study conducted in parallel with the JDIP attenuated vaccine studies, but using calves, has extended the findings in goats with similar results (Park et al., 2014).

### Phase III: the goat trial

Data from this final trial in a ruminant host were used to rank the remaining vaccine candidates to determine the best attenuated vaccine. Logistic issues interfered with testing and two strains that showed promising results in mice (strains 320 and 321) were not evaluated in goats (Bannantine et al., 2014). A second goal of this phase III vaccine trial was to validate the goat challenge model originally proposed in 2007 (Hines et al., 2007). Trial was performed at the University of Georgia-Tifton Veterinary Diagnostic and Investigational Laboratory.

Eighty 2-month-old goat kids were separated into 8 groups with 10 kids in each group. Three groups were dedicated to controls (wild-type MAP K-10, PBS and Silirum® vaccine). The remaining 5 groups consisted of the MAP vaccine candidates. The animals in each group were housed in identical treatment rooms in a manner to reduce “pen effect.” Baseline blood and fecal samples were taken at the beginning of the study. As with the other trials, the investigators were blinded as to the identity of the experimental vaccines. The Silirum® vaccine was administered as a single dose and used according to manufacturer instructions. The study length was approximately 18 months with a full 12-month period post-challenge. Two doses of each mutant vaccine were administered as described (Hines et al., 2014). All vaccine and challenge doses were delivered orally by syringe in pasteurized commercial goat milk.

While none of the vaccines tested prevented MAP infection or eliminated fecal shedding in goats, the Δ*fabG2_2* vaccine strain (coded 329 in Table 1) did lower the incidence and severity of infection as measured by lesion score, tissue pathology, fecal culture and fecal PCR (Hines et al., 2014). The *fabG2_2* gene encodes a 3-ketoacyl reductase of MAP and the transposon is inserted in the C-terminal half of the gene (Settles et al., 2014). This mutant has recently been shown to have defects in intestinal and liver persistence (Settles et al., 2014). Additional preliminary studies suggest that this mutation may not be stable as it could not be confirmed by PCR analysis. The Δ*fabG2_2* vaccine strain did not outperform the commercial vaccine control in the goat trial. This result may be explained by delivery route of the attenuated strains vs. the commercial vaccine. Attenuated strains were delivered orally and the commercial vaccine was delivered subcutaneously. Ideally, the route of vaccination should have been identical, but a second objective was to validate the goat challenge model proposed by the AMSC (Hines et al., 2007). In hindsight, the route of vaccination should have been the same for all vaccines.

## Conclusions

Vaccination against Johne's disease has considerable potential as a key management tool to control disease and transmission in ruminant livestock. Specifically, the use of attenuated mutants provide several advantages that include stimulation of a broad cellular immune response, ease of delivery, built in adjuvant characteristics and comparative cost. Several lessons were learned from the three-phase JDIP vaccine project as discussed below, and provide a rational framework for testing future vaccines against Johne's disease.

First, the results suggest that single gene knockout strains may not represent optimal vaccine candidates for MAP given the need to satisfy the “Goldilocks rule”—where the level of attenuation has to be just right in order to survive or replicate in target cells and stimulate a protective immune response but without causing disease. Consistent with this, while MAP vaccine candidates evaluated in the current trials were single gene knockouts, recent studies showed that a double mutant constructed in *M. tuberculosis* (*ΔfbpAΔsapM* was highly attenuated in human macrophages and, intriguingly, more immunogenic than the single knockout (*ΔfbpA*) (Saikolappan et al., 2012). These data also suggest the importance of careful targeting of genes/proteins for deletion based on prior knowledge of their role in pathogenesis, immunogenicity, or pathogen survival *in vivo*. For instance, the rationale for construction of the *M. tuberculosis* (*ΔfbpAΔsapM* strain was that *ΔfbpA*, which lacks Ag85, had already demonstrated immunogenicity and protection in mice, but the additional deletion of the acid phosphatase gene (*ΔsapM*) enabled the host phagosome to mature (Saikolappan et al., 2012). A considered approach for MAP vaccine candidate development that target orthologs in *M. tuberculosis* that have shown promise in vaccine efficacy trials or are known to be highly immunogenic targets seems reasonable, but has its limitations. For instance, there is no corresponding ortholog to *sapM* in MAP (Li et al., 2005), and hence, construction of a double knockout involving that locus would not be possible.

We note that the MAP vaccines evaluated herein represented the most comprehensive screen of MAP attenuated mutants to date, and included vaccine candidates with prior evidence of attenuation with *in vitro* or *in vivo* model systems that were enrolled on a volunteer basis (i.e., investigators were broadly invited and those that volunteered to participate and submit candidate vaccines were enrolled). This appears a reasonable strategy to adopt since it provides a facile and transparent mechanism for benchmarking candidate vaccines to each other to help identify the most promising candidates. However, not all described or available candidate strains of MAP were enrolled in this trial. Notably, the recently described Δ*leuD*, which provides protection in a goat model of infection (Faisal et al., 2013a) was not submitted for evaluation in the current trials, and neither were a set of invasion mutants (Alonso-Hearn et al., 2008) which should be included in future investigations.

Our studies have highlighted several major lacuna in our understanding of *in vitro* correlates of vaccine immunity and strain attenuation in MAP. For instance, how long should a strain of MAP take to be killed by phagocytes to be considered attenuated? And what are the physiologic (e.g., doubling time) or immunological (e.g., cytokine profiles elicited) correlates of protective immunity for MAP that should be considered prior to advancing a vaccine candidate? These and other similar questions regarding *in vitro* correlates of protection remain and need to be the focus of future investigations.

Second, and perhaps more importantly, the rationale for the use of the macrophage-to-mouse-to-native host pipeline for MAP vaccine development needs to be reconsidered. The survival of MAP strains in cultured macrophages has been previously used as a criterion for the selection of candidate vaccines for further testing in animal models. For instance, transposon mutants of MAP that were attenuated in macrophages were also shown to be attenuated in mice (Scandurra et al., 2009). While this strategy has been applied for selection of vaccine candidates for numerous other pathogens, particularly those that impact humans, the lack of validated or well rationalized *in vitro* correlates of protection does not enable an accurate assessment of either attenuation or immunogenicity of a given candidate *in vivo*. This shortcoming is further exacerbated by the lack of context for the host immune system and the natural milieu, and the fact that only short-term attenuation can be measured in macrophages, even though these are long-lived cells *in vivo*, can remain viable for only 48-h or so in laboratory culture. And can only measure degrees of attenuation in a somewhat artificial system that does not predict protection (Lamont et al., 2014).

The results of our investigations raise important questions on whether mice are relevant or appropriate models for assessment of anti-MAP vaccines. It has been long recognized that the mouse is not a natural host for MAP, but rather employed as a model for cost and practical (e.g., availability of immunologic reagents) considerations that may not be of relevance to infection or immunity against MAP in the natural host. By not being a natural host, challenge and vaccine studies are made difficult since it is often not possible to consistently infect mice. Furthermore, culturing of MAP from intestinal tissues (the predominant site of infection in the natural ruminant host) is rarely achieved in mice, and successfully infected animals do not exhibit any of the typical clinical signs (e.g., diarrhea) that are associated with MAP infection in ruminants. In hindsight, this is not altogether surprising since MAP infection is primarily a chronic granulomatous infection of the intestinal tract, and given that recent studies suggest that the mouse is a poor model for inflammatory conditions in humans (Seok et al., 2013), mice are not very likely to represent good models for chromic inflammatory diseases such as JD in cattle either. This is also consistent with recent observations (Scandurra et al., 2010; Park et al., 2011) that suggest attenuation in macrophages and marginal protection in the mouse model may not be a good predictor for the ruminant host. In addition, it was previously noted that a lack of survival in bovine macrophages does not predict survival in goats. For instance, the *pknG* mutant was attenuated in macrophages and yet showed persistence in goats and the immune response elicited by the mutant did not affect colonization by the MAP challenge (Park et al., 2011).

Thankfully, unlike the situation in humans, Johne's disease investigators have the luxury working with the natural (ruminant) host despite the apparent high costs. We suggest that the ability to quickly and relatively inexpensively use macrophage/mouse models, though tempting, may represent a situation of being “penny wise and pound foolish” and in the absence of clearly articulated and validated correlates of immune protection in these models, may result in perfectly good candidates being discarded during the triage process or, as was observed in the studies described herein, apparently promising targets move forward at considerable expense, only to fail to perform as expected when evaluated in the natural host.

In sum, the results of our studies strongly suggest that future investigations of MAP vaccine candidates are best conducted in the natural host, and considerable efficiencies can be realized by using a coordinated approach and standardized protocols for comparative benchmarking and evaluation of MAP vaccine candidates.

### Conflict of interest statement

The authors declare that the research was conducted in the absence of any commercial or financial relationships that could be construed as a potential conflict of interest.
